# Common beliefs about sexuality: a Tunisian survey among pregnant women

**DOI:** 10.1192/j.eurpsy.2023.1338

**Published:** 2023-07-19

**Authors:** A. Zouari, F. Guermazi, B. Amamou, S. Hentati, I. Feki, I. Baati, L. Gaha, J. Masmoudi

**Affiliations:** 1 psychiatry A, Hedi Chaker Hospital, sfax; 2 psychiatry, EPS Fattouma Bourguiba, monastir, Tunisia

## Abstract

**Introduction:**

Sexual relationship is affected by the emotional factors, changes in women’s body, sexual dysfunctionsand also common beliefs about sex in pregnancy. Couples might tend to avoid sexual intercourse in pregnancy due to different beliefs.

**Objectives:**

Our objective was the evaluation of sexual related and common beliefs among pregnant womens.

**Methods:**

We conducted a cross-sectional and descriptive study. We targeted Tunisian pregnant women whatever the term of pregnancy. We collected data using a self-questionnaire performed with Google Forms and posted regularly on social networks over a period of six months. In order to assess the most common beliefs, we used a panel of questions inspired by the literature.

**Results:**

34 pregnant women participated to the study. Mean age was 31,56 years (SD =3,25). All the participants had a secondry or university education (5.9% and 94.1% respectively). Ninteen women (55.9%) were multiparous with 44% in the third trimester.

Among participants, 20.6% believe that sexual intercourse can be harmful to the baby, 41.2% believe that the number of intercourse should be limited during pregnancy and 17.6% thought that sexual intercourse should be stopped in the first three months.

In relation to body image, 11.8% of women approve that pregnancy takes women’s all beauty and 8.8% thought that their bodies weren’t attractives as before for spouses. About a quarter of our population (23.5%) agreed that pregnant women lose sexual desires and 14.7% approved that intercourse satisfies only men. Only one women (2.9%) reported that intercourse during pregnancy is considered a sin.

**Conclusions:**

We have identified through this study different beliefs about sexuality during pregnancy, sometimes aberrant and which can affect the sexuality of couples. Attention of health professionals should be attracted to this issue and sexuality should definitely be integrated into prenatal care and counselling.

**Disclosure of Interest:**

None Declared

